# Bridging gaps in medication therapy management at community health centers: A mixed-methods study on patient perceptions and pharmacists' preparedness

**DOI:** 10.1016/j.rcsop.2024.100554

**Published:** 2024-12-16

**Authors:** Nanang Munif Yasin, Fivy Kurniawati, Firda Ridhayani

**Affiliations:** a Department of Pharmacology and Clinical Pharmacy, Faculty of Pharmacy, Universitas Gadjah Mada, Yogyakarta, Indonesia; b Department of Pharmacology and Therapeutics, Faculty of Medicine, Public Health, and Nursing, Universitas Gadjah Mada, Yogyakarta, Indonesia

**Keywords:** Pharmacist, Practice, Primary healthcare, Professional

## Abstract

**Background:**

The primary goals of Medication Therapy Management (MTM) are to avoid pharmaceutical mistakes, facilitate accessible therapy, and encourage patients to actively participate in their health management.

**Objectives:**

This study aimed to determine patients' perceptions of MTM services, evaluate the knowledge, attitudes, and practices (KAP) of Community Health Center (CHC) pharmacists regarding MTM services, and develop strategies to improve MTM services in CHCs.

**Methods:**

A mixed-method approach was designed in three parts. First, a study was conducted with diabetic or hypertensive patients at CHCs around Yogyakarta to assess their perceptions of MTM elements and benefits. Second, a survey was conducted among CHC pharmacists to determine their KAP concerning MTM and the current service provided. In the third part, findings from the second stage were used to establish appropriate MTM services for CHCs through focus group discussions (FGDs) and to explore obstacles to implementation. Qualitative data were analyzed using inductive content analysis.

**Results:**

Among 117 patient participants, over 60.0 % perceived positive benefits from five elements of MTM. Among 37 pharmacist participants, 19 (51.4 %) of them did not understand MTM concepts, with 14 (37.9 %) pharmacists demonstrating a low level of knowledge. Nearly all pharmacists held a positive attitude towards MTM. Time constraints were identified as barriers to MTM implementation. Based on the FGD with 24 pharmacists, three main themes and ten sub-themes were identified.

**Conclusions:**

MTM services have not been fully implemented by pharmacists at CHCs. Future implementation of MTM is expected to be more adaptive to the CHCs condition, integrated with existing systems, standardized in terms of procedures and facilities.

## Highlights

• The practice of MTM services is not widespread across Indonesia.
• Pharmacists' KAP on MTM services is essential for establishing suitable MTM model.
• Most of the patients perceived positive benefits from five elements of MTM.
• Future implementation of MTM is expected to be more adaptive to the CHCs condition.

## List of abbreviations

**Table:** 

CHCs	Community health centers
COREQ	Consolidated criteria for reporting qualitative research
CPP	Cumulative patient profile
FGD	Focus group discussion
KAP	Knowledge, attitude, and practice
MAP	Medication-related action plan
MTM	Medication therapy management
MTR	Medication therapy review
PHC	Primary healthcare
PMR	Personal medication record
STROBE	Strengthening the Reporting of Observational Studies in Epidemiology

## 1 Background

Drug-related problems continue to be a major public health concern worldwide.^1^ Data from the United States indicate that there are around 1.5 million preventable adverse drug events annually, leading to an additional $3.5 billion in healthcare costs and 98,000 deaths.^2^ To prevent drug-related issues, regulations mandate that health services be prompt, efficient, patient-focused, safe, effective, and tailored to patient needs. They also require patient involvement in the process.^3^

Medication therapy management (MTM) is a service model designed to address overall health issues by reducing treatment-related morbidity and mortality, as well as optimizing therapeutic outcomes for individual patients.^4^, ^5^, ^6^ MTM services have demonstrated improvements in both adherence and glycemic control among underserved patient populations.^7^ This model is a specialized service or range of services that enhances therapeutic results by prioritizing active patient participation and improving collaboration between healthcare providers. The implementation of MTM model refers to five core elements: a comprehensive review of a patient's medications to address medication-related issues (Medication Therapy Review = MTR), maintaining a detailed medication list for appropriate use (Personal Medication Record = PMR), providing a patient-focused action plan for medication management (Medication-Related Action Plan = MAP), pharmacist interventions or referrals to other healthcare providers (Intervention and/or Referral), and continuous documentation with follow-up to monitor patient progress (Documentation and Follow-Up).^8^

The primary goals of MTM are to avoid pharmaceutical mistakes, facilitate accessible therapy, and encourage patients to actively participate in their health and medication management.^9^^,^^10^ Pharmaceutical mistakes, including incorrect medication, treatment duplication, drug interactions, and contraindications, can lead to adverse drug reactions. Several studies have reported evidence of the potential and value of pharmacists-led MTM services, which successfully enhances patients' adherence to medication and improves their clinical outcomes.^10^, ^11^, ^12^ This model emphasizes the crucial role of collaboration between healthcare providers and patients in improving medication adherence, clinical outcome for chronic diseases, and minimizing drug-related adverse events.^13^^,^^14^

Pharmacists, general practitioners, nurses, and allied health professionals exclusively comprise the primary healthcare (PHC) or community health center (CHC) team. The PHC services primarily assist in the management of long-term health care, including chronic conditions like diabetes, with an emphasis on preventative and promotional initiatives to attain a high standard of community health.^15^^,^^16^ In Indonesia, primary healthcare serves as the first point of contact and gatekeeper in responding to health issues.^17^^,^^18^ Thus, the implementation of MTM in primary healthcare is crucial. Nevertheless, due to a lack of pharmacists familiar with the concept of MTM services, the practice of MTM services is not widespread across Indonesia.

The aims of this study were to determine patients' perceptions regarding MTM services, evaluate CHCs pharmacists' knowledge, attitudes, and practices (KAP) concerning MTM services, and develop strategies to improve MTM services in CHCs. Furthermore, it also identified obstacles and challenges upon MTM implementation, which are essential to address as part of an initial strategy for establishing an appropriate MTM model to optimize pharmaceutical care services in Indonesia.

## 2 Methods

This study comprised three stages: 1) Stage 1: a survey on patients' perceptions of PHC regarding MTM elements and their benefits; 2) Stage 2: a survey on the KAP of CHCs pharmacists concerning MTM and the current forms of service provided; and 3) Stage 3: a Focus Group Discussion (FGD) on an appropriate MTM model for CHCs and the obstacles to its implementation.

Ethics approval for this study was granted by the Medical and Health Research Ethics Committee, Faculty of Medicine, Public Health, and Nursing, Universitas Gadjah Mada, Indonesia, under the letter number KE/FK/1879/EC/2023. All participants provided informed consent before participating in the study.

This study adheres to The Strengthening the Reporting of Observational Studies in Epidemiology (STROBE) Statement and the Consolidated Criteria for Reporting Qualitative Research (COREQ) checklists.^19^^,^^20^

### 2.1 Stage 1: survey on patients' perceptions of MTM elements and their benefits

#### 2.1.1 Study design and reporting

This research employed a multicenter cross-sectional design, using questionnaires distributed to patients receiving treatment at eight CHCs in Yogyakarta. The survey focused on patients' perceptions of the five main elements of MTM and the benefits of MTM services at CHCs.

#### 2.1.2 Setting, subject of the study, and sampling technique

Patients were recruited using a consecutive sampling method from eight primary healthcare centers in Yogyakarta, Indonesia. After agreeing to participate in this study, patients were briefed on MTM-related terminology and guided in completing the questionnaire. All patients were briefed both verbally by the researcher and through written materials provided in the study explanation form regarding the concept and elements of MTM. Throughout the questionnaire completion process, a minimum of two researchers assisted each patient in answering any questions related to the questionnaire. All patients who participated in this study were aged 18 years or older, with a diagnosis of type 2 diabetes or stage 1 hypertension. The sample size was determined using the Lemeshow formula, requiring at least 96 patients.^21^

#### 2.1.3 Instrument and data collection

A self-report questionnaire was used to obtain patients' sociodemographic data (gender, age, level of education, employment, family history, comorbidity, and number of medications) and their perception of MTM implementation. The questionnaire included six questions about perceptions of MTM and four about the perceived benefits of MTM, with two response options: yes and no. This questionnaire was developed and administered to 30 patients for validation, covering readability and usability. All patients were able to complete all questionnaire items without difficulty. The questionnaire was distributed over four months in CHCs in Yogyakarta.

#### 2.1.4 Data analysis

Data analysis used descriptive statistics to determine patients' characteristics and their perceptions regarding MTM. SPSS version 22 was used to analyzed these data.

### 2.2 Stage 2: survey on KAP of CHC pharmacists regarding MTM and the current forms of service provided

#### 2.2.1 Study design and reporting

An observationalapproach was used, comprising a multicenter cross-sectional study followed by structured interviews with pharmacists. The multicenter cross-sectional study aimed to identify pharmacists' KAP regarding MTM implementation and to obtain a picture of the current services provided. This study adhered to the STROBE Statement.^20^

#### 2.2.2 Setting, subject of the study, and sampling technique

This study was performed among pharmacists in CHCs around Yogyakarta, Indonesia. Pharmacists were recruited using consecutive sampling methods from all CHCs in Yogyakarta, Indonesia. The study participants were pharmacists involved in pharmacy services at their workplaces.

#### 2.2.3 Instrument and data collection

The questionnaire was adapted from an earlier validated questionnaire, from Al-Tameemi and Sarriff (2019) study, and developed based on the KAP theory, divided into three parts.^10^ The first part consisted of ten questions about pharmacists' knowledge related to MTM services. These questions were true/false questions. Each correct answer was given “1” point while wrong answer was given “0” point. The second part included six positive statements and 5-point Likert scale was used to assess the attitude. Each question contained five statements and the rating scale was measured as follows: positive statement with choices strongly agree, agree, uncertain, disagree, and strongly disagree, and scores 5, 4, 3, 2, and 1, respectively regarding pharmacists' attitudes towards MTM services. The final part comprised several questions pertaining to pharmacists' practices and using MTM services in their workplaces. This section consisted of 12 questions to assess the practice of pharmacists on MTM service, the compliance with the principle of MTM service and state the barriers that might affect providing MTM service in hospital setting. All questions were Yes/No questions. No answer was given “0” point and Yes answer was given “1” point. This questionnaire was validated by three clinical pharmacy lecturers for content relevancy and suitability, and was tested for reliability among 15 pharmacists at CHCs for knowledge, attitude, and practice with score of Cronbach's alpha of 0.326, 0.716, and 0.822, respectively.^10^ In addition to KAP, a survey was conducted to identify the types of services provided by pharmacists at CHCs, using open-ended questions. This self-reported questionnaire was distributed over two months in CHCs in the Yogyakarta Special Region.

#### 2.2.4 Data analysis

Descriptive sociodemographic characteristics (gender, age, latest education level, and duration of practice) were displayed as percentages. SPSS version 22 was used to analyzed these data. Knowledge, attitudes, and practices related to MTM services among pharmacists were described using percentage data.

### 2.3 Stage 3: Innovative MTM model in CHCs: Findings from focus group discussion - Qualitative study

#### 2.3.1 Study design and reporting

This study employed a qualitative technique. The findings are reported following the COREQ checklists.^19^

#### 2.3.2 Setting, subject of the study, and sampling technique

Pharmacists were invited through CHCs using purposive sampling to attend FGD in the Faculty of Pharmacy, Universitas Gadjah Mada, Yogyakarta. Inclusion criteria included any pharmacists in CHCs around Yogyakarta, Indonesia.

#### 2.3.3 Instrument and data collection

All pharmacists were informed about the study by professional teams (NMY, FK, and FR). Before conducting the FGD, invited pharmacists were asked to complete a questionnaire about their readiness to implement MTM. The FGD topic guide was created based on the questionnaire responses and literature. The FGD guide was used to stimulate discussion, and participants were encouraged to express relevant MTM service issues. The semi-structured and face-to-face FGD was conducted to explore the barriers to implementing MTM and the most feasible MTM model for public health centers. The FGD was conducted and moderated by NMY, a male lecturer with expertise in pharmacy services development (PhD), accompanied by two facilitators, FK and FR, and lasted about 90 min. The FGD was held once in a large group involving 24 pharmacists. The session was structured into four segments: a 10-min introduction, a 35-min discussion on experiences in delivering pharmaceutical services, a 35-min discussion on expectations and recommendations for future service models, and a 10-min conclusion. Each pharmacist was provided with the opportunity to contribute both in writing and verbally during the FGD session. Data saturation was achieved when no new information emerged, and the data did not alter the research findings.

The moderator leads the FGD with the following sequence: introduction, including an overview of the FGD (purpose and process guide) and participant self-introductions; a discussion of experiences in providing pharmaceutical services (types, challenges, etc.); expectations and suggestions for future service models; and concluding the discussion (summarizing, additional comments or questions). The moderator's role is to guide the FGD according to the discussion guide, ensuring each participant has the opportunity to share their views. Once a topic is sufficiently explored and no new ideas are raised, the moderator moves on to the next topic.

During data collection, only participants, researchers, and facilitators were present. Participants were encouraged to communicate openly and were followed up with non-directive universal inquiries. The FGD session was not repeated for all participants. Sessions were audio-recorded, fully transcribed, and verified for accuracy by all researchers. The transcripts were not returned to the respondents for feedback or revision. During the FGD session, the researchers did not record the participants' reactions on field notes. Researchers and FGD moderators had no prior relationships with participants and initiated each session with a brief overview of the research team and the rationale behind the study.

#### 2.3.4 Data analysis

The data were analyzed using inductive content analysis by NMY and FK. The analysis process was as follows: 1) reading and reviewing the original scripts repeatedly to ensure an overall comprehension, 2) formulating keywords and encoding, 3) categorizing the data according to the codes, 4) formulating categories and defining major themes based on information obtained from transcripts. The data analysis process in this qualitative research involved in-depth discussions among the research team until consensus was reached on key themes, interpretations, and potential biases, ensuring that multiple perspectives are considered and minimizing individual bias. In this study, two researchers conducted separate analyses, each reviewing the relevant items independently. Afterward, the three researchers convened multiple times to discuss and reach a consensus on the emerging themes and applied codes. In cases where discrepancies arose between the initial evaluations, a third researcher acted as a mediator to help resolve the differences and ensure agreement. This process ensured that the analysis was conducted in a thorough and consistent manner.

## 3 Results

From the questionnaires distributed to patients at eight CHCs in Yogyakarta, 117 patients completed them fully. The patient characteristics were predominantly female (70.9 %, *n* = 83) compared to male (29.1 %, *n* = 34). Additionally, 42.7 % (*n* = 50) were aged 65 or older, while 57.3 % (*n* = 67) were aged 18–64. Among the patients, 60.7 % (*n* = 71) had comorbidities, and 38.4 % had a family history of disease, including hypertension (16.2 %, *n* = 19) or diabetes mellitus (22.2 %, *n* = 26).

**Fig. 1:**
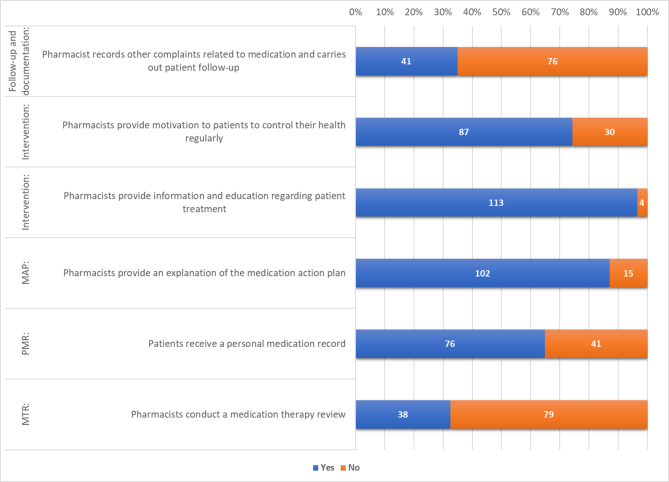
Patient perception of MTM core element. Notes: “Yes” indicates that the patient agrees with the questionnaire statement. “No” indicates that the patient does not agree with the questionnaire statement.

Patients' experiences with the five elements of MTM are summarized in Fig. 1. Over 60.0 % of patients reported that pharmacists provided PMR, MAP, and interventions, while approximately 33.0 % of patients indicated that pharmacists also conducted MTR and documentation as part of the MTM process.

**Fig. 2:**
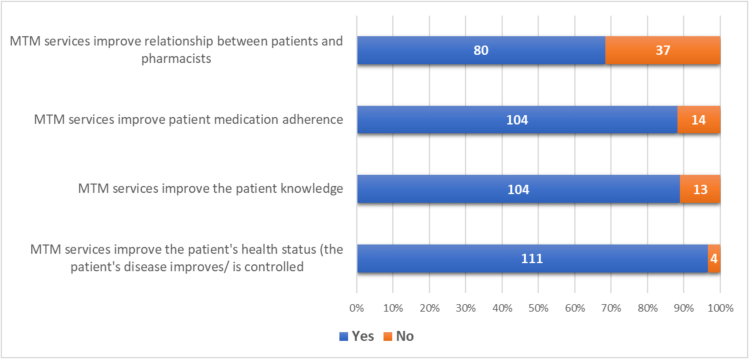
Patient perceptions on the benefits of MTM. Notes: “Yes” indicates that the patient agrees with the questionnaire statement. “No” indicates that the patient does not agree with the questionnaire statement.

As shown in Fig. 2, the majority of patients experienced benefits from MTM. More than 80.0 % of patients perceived that MTM improved their health status, adherence, and knowledge, while only about two-thirds felt that it fostered a good relationship with the pharmacist.

A total of 37 pharmacists from primary healthcare centers in Yogyakarta, Indonesia, agreed to contribute to the study, with 24 of them participating in stage 2. The sociodemographic details of the participants are displayed in Table 1. The participants' mean age was 38 years, and the majority of pharmacists (86.5 %, *n* = 32) were female. It was observed that only 8.1 % (n = 3) of participants had a master's degree and most had been practicing pharmacy services in primary healthcare for more than 10 years (51.4 %, *n* = 19). In Indonesia, the qualification process for becoming a pharmacist typically involves completing a five-year pharmacy program at a recognized university. This program begins with a bachelor's degree and continues with a professional pharmacy degree in the fifth year, which includes both theoretical coursework and practical training.

**Table 1:** Sociodemographic characteristics of pharmacists.

	Stage 1 (*n* = 37)		Stage 2 (*n* = 24)	
Characteristics	n	%	n	%
Gender				
Male	5	13.5	1	4.2 %
Female	32	86.5	23	97.8 %
Age (years)				
18–30	4	10.8	2	8.3 %
31–45	29	78.4	20	83.4 %
>45	4	10.8	2	8.3 %
Educational background				
Pharmacist professional degree	34	91.9	22	91.7 %
Master	3	8.1	2	8.3 %
Years of practice				
1–5	11	29.7	6	25.0 %
6–10	7	18.9	4	16.7 %
>10	19	51.4	14	58.3 %

All participants completed the questionnaires. The first part consisted of ten different items used to assess the knowledge of pharmacists in primary healthcare settings related to MTM service. Each question was worth 1 point, with a total of 10 points for all questions. The pharmacist's level of knowledge is reported in this study. Unfortunately, more than one third of pharmacists were found to have a low level of knowledge, pharmacist’ knowledge score ranged from 0 to 5, due to insufficient understanding of the MTM service (*N* = 14, 37.9 %), while the other pharmacists have moderate and high levels of knowledge, at 29.7 % (*N* = 11) and 28.0 % (*N* = 12), respectively. At a high knowledge level, the pharmacist's knowledge score ranged from 8 to 10, while at a moderate knowledge level, the pharmacist's knowledge score ranged from 6 to 7. The aspects of knowledge that were the least understood by pharmacists were mainly the definition of MTM, the active contribution of the patient, the main role of MTM services, and the core elements of MTM, at 64.9 %, 70.3 %, 48.6 %, and 51.4 %, respectively (Table 3).

Apart from knowledge aspects, this study also assessed pharmacists' attitudes towards MTM service (Table 2). The majority of participants showed a positive attitude. All participants believed that their main role as pharmacists goes beyond dispensing medication. Furthermore, through MTM service, they were convinced that patients would acquire sufficient and valuable guidance about their chronic illness and medication.

**Table 2:** Pharmacists' knowledge, attitude, and practice towards MTM service and barriers of its implementation.

Questions	(n = 37)	
	Positive Answer	Negative Answer
Knowledge Aspects		
MTM definition	13 (35.1 %)	24 (64.9 %)
Goals of MTM services	37 (100 %)	0 (0.0 %)
Core elements of MTM services	18 (48.6 %)	19 (51.4 %)
Patients who could potentially benefit from MTM services	25 (67.6 %)	12 (32.4 %)
Primary role of MTM services	19 (51.4 %)	18 (48.6 %)
MTM helps optimize pharmacists' role	33 (89.2 %)	4 (10.8 %)
MTM can be applied by either pharmacists or pharmacy technicians	20 (54.0 %)	17 (46.0 %)
MTM require active contribution from patients	11 (29.7 %)	26 (70.3 %)
MTM could reduce patients' visit and cost among patients with chronic condition	31 (83.8 %)	6 (16.2 %)
MTM could enhance patients' satisfaction	36 (97.0 %)	1 (3.0 %)

Attitude Aspects		
Appraising patient's medication profile and delivering treatments are important duties for pharmacist to prevent adverse drug events	37 (100.0 %)	0 (0.0 %)
MTM implementation through healthcare providers would provide patients with adequate and valuable information about their disease	37 (100.0 %)	0 (0.0 %)
MTM service is favorable considering the five fundamental elements of MTM service	37 (100.0 %)	0 (0.0 %)
Providing MTM services is an unprecedented opportunity for pharmacists to make a greater contribution to patient care	37 (100.0 %)	0 (0.0 %)
Compared to other healthcare professionals, patients' therapeutic outcomes would be enhanced if medications were closely monitored by a pharmacist	33 (89.2 %)	4 (10.8 %)
MTM service implementation requires broader knowledge than basic pharmacy practice expertise	29 (78.4 %)	8 (21.6 %)

Practice Aspects and Barriers		
Desire to be an MTM provider in the future	36 (97.0 %)	1 (3.0 %)
The critical role of MTM services in improving health quality	37 (100.0 %)	0 (0.0 %)
Desire to learn in detail how to deliver MTM service	37 (100.0 %)	0 (0.0 %)
Time allocation in health care for doing regular tasks	32 (86.5 %)	5 (13.5 %)
Offline courses method for pharmacist training	29 (78.4 %)	8 (11.6 %)
Lack of training is one of the possible obstacles to utilizing MTM services	32 (86.5 %)	5 (13.5 %)
Availability of private counseling room in the workplace	18 (48.6 %)	19 (51.4 %)
Accessibility to guidelines and drug information resources, either online or in a printed version	21 (56.8 %)	16 (43.2 %)
Routine access to the most updated treatment guidelines, either online or in hard copies	19 (51.4 %)	18 (48.6 %)
Online education is an effective way to assist pharmacist training	19 (51.4 %)	18 (48.6 %)
Having sufficient time in the future to utilize MTM services	13 (35.1 %)	24 (64.9 %)
Adopting MTM services needs high-cost	8 (25.6 %)	29 (78.4 %)

Abbreviations: MTM, medication therapy management.

As shown in Table 3, the results of pharmacists' practice towards MTM service and obstacles to its implementation showed that every pharmacist believed that MTM service would enhance the quality of healthcare services and they were eager to acquire in-depth information regarding MTM service. About half of the pharmacists (51.4 %, *n* = 19 %) agreed that an online education scheme is an excellent approach to training for pharmacists about MTM service, while most of them (78.4 %, *n* = 29) preferred to be involved in offline training workshops regarding MTM service. Despite their belief in MTM implementation, pharmacists identified several potential obstacles that might affect MTM service implementation, with the most significant challenges being the high cost involved (78.4 %), the lack of private counseling rooms (51.4 %), and the lack of time to apply MTM service (64.9 %).

**Table 3:** Theme and quotes related to MTM implementation in CHCs.

Sub Theme	Representative quote (participant ID)
Theme 1: Adaptive MTM Model Suitable for the Conditions of CHCs	
Sub Theme 1 A simple and easy-to-implement service model	“A service model that can be implemented without being burdensome in documentation and would be even better if it is integrated into the CHC system.” (Pharmacist 1)
	“A simple service model that does not require many tools or extensive documentation, as pharmacy staff are very limited and the current workload already takes up a lot of time.“(Pharmacist 24)
	“An MTM model that simplifies services without adding to the workload and will improve the quality of service.” (Pharmacist 17)
	“A simple and easy-to-implement service, so it can be used by all pharmacy staff and sustained in the long term.“(Pharmacist 14)
Sub Theme 2 A comprehensive service model integrated into the community health center's management information system	“MTM should be conducted electronically and integrated with the CHC management information system.“(Pharmacist 10)
	“There should be a system integrated with the CHC management information system to facilitate recording and documentation.” (Pharmacist 5)
	“The availability of comprehensive tools that are easy for pharmacists and other healthcare workers to use.” (Pharmacist 7)
	“An MTM model that is practical and comprehensive, with integrated but efficient documentation.” (Pharmacist 13)
Sub Theme 3 A collaborative service model	“It should be implemented through cooperation among healthcare workers or integrated with other healthcare professionals.” (Pharmacist 19)
	“MTM should be implemented collaboratively with other healthcare workers at CHCs and also involve the community independently.” (Pharmacist 6)
	“A form or system should be created that is integrated with doctors and other healthcare to easily create PMR, MAP, and other documentation.” (Pharmacist 8)

Theme 2: Standardized MTM services in CHCs	
Sub Themes	Representative quote
Sub Theme 1 The need for special training to understand MTM	“Training should be conducted before MTM is practiced in CHC.” (Pharmacist 3)
	“There should be specialized MTM training for pharmacists with dedicated MTM training modules.” (Pharmacist 22)
	“Intensive socialization and training on MTM for staff are necessary.” (Pharmacist 20)
Sub Theme 2 The need for standard facilities and instruments	“Services should be equipped with standardized forms that can be filled out quickly and easily understood, possibly in the form of a checklist, which can be used directly or indirectly.” (Pharmacist 17)
	“CHC should have the facilities to implement MTM, such as a dedicated counseling room, computers, and specialized MTM service modules.” (Pharmacist 11).
Sub Theme 3 Recording and documentation system	“In the pharmacy section, it is difficult to find files because patients come at any time. There is an electronic information system now, but there is no menu for patient medication notes. It is suggested to create this menu to make counseling easier.” (Pharmacist 18).
	“Patients are given a card from the pharmacy to show during check-ups. During consultation with the doctor, additional notes are made. Ideally, it would be like a book, but this has not yet been implemented.” (Pharmacist 2)
	“Prescriptions are still manual. There is a pharmacy confirmation book (containing mixed information). The most effective way would be online (electronic information system). Data retrieval would also be easier (just download).” (Pharmacist 17)
	“PMR storage is not yet neat or well-organized. It is difficult to find the history of previous visit sheets.” (Pharmacist 10)
Sub Theme 4 Facilities and infrastructure.	“The room is small (inadequate), limiting it to only providing medication information. We are just starting to initiate PMR and create PROLANIS cards.” (Pharmacist 5)
	“Documentation is not yet optimal or orderly. Recording is not neat. Documentation is still on the back of the prescription sheet signed by the pharmacist.” (Pharmacist 21)
	“There are many patients, so pharmaceutical services are limited to monitoring medication use, side effects if any, and education for routine control, which has not been comprehensively recorded due to difficulties in documentation.” (Pharmacist 10)

Theme 3: Factors influencing the implementation of MTM in CHCs	
Sub Themes	Representative quote
Sub Theme 1 Allocation of specific time	“The staff have too much workload, making it difficult to focus on providing ideal MTM.” (Pharmacist 2)
	“The number of healthcare workers is limited during routine services, so they cannot specifically monitor patients' medication.” (Pharmacist 27)
	“Communication time with patients is limited, so special services are only provided if patients actively raise complaints.” (Pharmacist 9)
Sub Theme 2 Pharmacists' competence	“There is a lack of skill in writing notes on counseling sheets and other forms.” (Pharmacist 8)
	“Recording and documentation are still done according to personal preference because I do not yet know how to make good notes. Doctors have a SOAP note format. Therefore, training on how to write like doctors is needed.” (Pharmacist 10)
Sub Theme 3 A supporting system	“Advocacy to policymakers is needed for the implementation of MTM.” (Pharmacist 15)
	“There should be adequate human resources and infrastructure support for the implementation of MTM services.” (Pharmacist 4)
	“Support from all parties, especially management, is necessary.” (Pharmacist 9)

Abbreviations: CHC, community health center; ID, identity; MTM, medication therapy management; SOAP, subjective objective action plan.

The MTM elements most frequently implemented by pharmacists in CHCs are, in order, interventions/referrals (86.5 %), PMR (67.6 %), MTR (54.1 %), documentation and follow-up (51.4 %), and MAP (35.1 %). The types of activities of MTR implemented by pharmacists in CHCs included prescription assessment and services, direct assessment of patients regarding medication, lab results, medication intake, and lifestyle (during prescription screening, counseling documentation, and form filling).

Implementation of the PMR included various forms such as a patient medication pick-up sheet that contained the patient's name, date of pick-up, and signature, along with columns for counseling, drug-related problems and adverse drug reactions. It could also be in the form of a pocketbook that included notes on lab results, and a cumulative patient profile (CPP) form. Additionally, a dynamic card could be used, which contained the control date, lab results, and provided therapy.

Moreover, other elements of MTM, such as the MAP, were documented in a consultation form for pharmacists working with other healthcare professionals (doctors, nurses, lab, and medical records) and in both the consultation book and patient medication record.

Activities that have been implemented as part of the intervention and referral element included corrections to prescription writing, adjustments to dosage, and drug selection. Adverse drug reaction reports were submitted to doctors, ensuring prompt attention and management. Furthermore, interventions were made with patients to provide guidance on the proper method and timing of medication intake, aimed to improved adherence and therapeutic outcomes.

Moreover, several activities related to follow-up and documentation have been implemented at the CHCs, included the use of a communication book with doctors, a counseling documentation book, and a special consultation book for medication, which is used in collaboration with other healthcare professionals. This consultation book is signed by a doctor, dentist, or midwife, as well as a pharmacist, ensuring proper documentation and coordination of care.

The most successfully implemented MTM element is interventions/referrals (86.5 %), which includes correcting prescription writing, adjusting dosages, selecting medications or managing adverse drug reactions communicated to doctors, and advising patients on medication usage and timing. The weakest MTM element is the MAP (35.1 %), which involves consultation forms and patient consultation books and medication records. The other elements, documentation, MTR, and PMR, have been implemented by approximately half of the pharmacists.

**Fig. 3:**
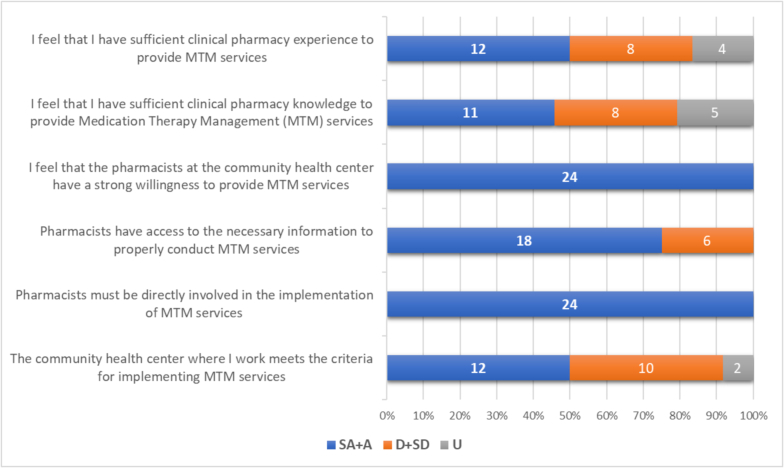
Pharmacists' readiness for MTM implementation. Notes: SA = Strongly Agree; A = Agree; D = Disagree; SD = Strongly Disagree; U = Uncertain.

Among the 37 pharmacists who participated at stage 2, only 24 pharmacists attended and were involved in the FGD. Regarding the readiness for MTM implementation, all pharmacists agreed that they were willing and needed to be directly involved (Fig. 3). Approximately half of the pharmacists felt prepared to perform MTM in terms of knowledge (45.8 %) and experience (50.0 %), and they were confident that their institution could implement MTM (50.0 %).

Based on the FGD data, three main themes and ten sub-themes were identified (Table 3). The main themes are an adaptive MTM model suitable for the conditions of CHCs, standardized MTM services, and the factors influencing the implementation of MTM in CHCs.

An adaptive MTM model suitable for the conditions of CHCs was identified as a main theme. The adaptive MTM model refers to an approach that is tailored to the specific healthcare context in Indonesia, considering the limited number of pharmacist staff and the lack of an integrated healthcare system. This model requires adjustments to the standard MTM practices to ensure they are feasible and effective within the existing infrastructure. The sub-themes include a simple and easy-to-implement service model, a comprehensive service model integrated into the CHCs' management information system, and a collaborative service model.

Standardized MTM services were identified as the second main theme. The sub-themes include the need for special training to understand MTM, the need for standard facilities and instruments, a recording and documentation system, facilities and necessary infrastructure.

Factors influencing the implementation of MTM were identified as the third main theme. The sub-themes include the allocation of specific time, pharmacists' competence, and a supporting system.

## 4 Discussion

Previous studies in the United States, specifically from Maryland and Delaware, have shown that 80–86 % of patients have never received components of MTM services, such as MTR, PMR, and MAP.^22^ Another study from California found that 93 % of respondents were unaware of MTM services.^23^ The current research findings align with these studies, as nearly two-thirds of the respondents had not received MTR, follow-ups, or documentation. Similarly, about 50 % of pharmacists in CHCs do not perform MTR, follow-ups, or documentation.

However, a positive outcome from this study is that more than two-thirds of patients reported that the pharmaceutical services provided benefits related to their health status, adherence, knowledge, and the existence of the pharmacist's role. These findings are similar to those by Truong et al. (2006), where three-quarters of patients reported positive perceptions of MTM's impact on health, medication use, communication, and their relationship with pharmacists.^22^

In terms of the five elements of MTM, patients and pharmacists in CHCs identified MTR and documentation as areas needing improvement, while PMR, MAP, and interventions also require enhancement. Increasing the involvement of CHCs pharmacists in MTM services should further evaluate patients' perceptions of MTM's value. Greater efforts are needed to raise public awareness of MTM and its benefits.

A comprehensive assessment of pharmacists' KAP regarding their role as health service providers is expected to form the foundation for developing an MTM adaptation framework for primary healthcare in Indonesia.^24^ MTM is a recent model extending pharmacists' services beyond traditional roles, engaging both patients and other healthcare workers to achieve better therapeutic outcomes, particularly for chronic disease management.^10^^,^^25^

The successful implementation of MTM as a pharmacist-led program requires assessing pharmacists' KAP to ensure professional readiness. Understanding their KAP as primary MTM providers is critical for identifying necessary interventions to improve patient medication adherence.^10^^,^^26^ According to this survey, 37 % of pharmacists had low awareness of MTM, although more than 70 % held positive attitudes towards MTM, despite various barriers.

Most pharmacists in this survey had limited understanding of MTM, whereas previous studies from Malaysia and Saudi Arabia reported higher levels of MTM literacy among pharmacists.^10^^,^^27^, ^28^, ^29^ In Malaysia, they regularly practice in medication therapy adherence clinics, a service nearly similar to MTM. This allows them to have close interactions with patients and deliver direct patient assistance such as counseling, providing patients with further pharmacological information concerning their conditions and treatments, and improving patients' adherence to their medications.^10^ Consequently, they are equipped with adequate knowledge specifically for MTM through their regular practice activities, unlike Indonesian pharmacists, who are only familiar with basic information without understanding the underlying concepts of MTM. Therefore, pharmacists in this study must be educated on MTM services, including their purpose, core elements, and the importance of active patient involvement. This education will enhance their ability to deliver optimal care, especially in improving patients' knowledge and medication adherence. In Indonesia, pharmacists have the opportunity to engage in a variety of educational programs provided by the Ministry of Health and academic institutions, including seminars, workshops, and expert discussions to gain in-depth knowledge related to MTM.

In practice, all pharmacists expressed willingness to engage in MTM and believed that it would improve the quality of health services. However, challenges remain, such as high workload, limited time, lack of counseling rooms, and insufficient MTM training. These challenges mirror findings from American research, where pharmacists cited similar barriers to MTM adoption.^9^^,^^30^

Adequate time is crucial for pharmacists to thoroughly address patient therapy problems and provide tailored advice. Counseling rooms are necessary for private consultations, where detailed discussions can take place.^31^ Addressing these barriers is vital to support the successful implementation of MTM services and improve patient outcomes.

This study also explored the readiness of CHCs to implement MTM through qualitative FGD. Most pharmacists were unfamiliar with MTM, and its implementation would increase their workload. An innovative and adaptable MTM model is needed to suit CHCs conditions. FGD results indicated that pharmacists desire an easy-to-implement MTM model with simple documentation that can be sustained long-term. Given their current workload, collaboration with other healthcare providers would ease the burden. Integrated forms with other healthcare workers would further facilitate MTM implementation. Similar to other studies, CHCs managers should review pharmacists' workloads and strengthen collaboration among healthcare workers, including access to medical information.^26^^,^^30^^,^^32^

Furthermore, MTM services must be comprehensive, with well-documented patient data accessible to all healthcare providers. CHCs in Yogyakarta have been implementing Electronic Health Records (EHR) using the SIMPUS application since 2022 to comply with Health Minister Regulation No. 24/2022, which requires full EHR implementation by December 2023. While pharmacists have access to patient medical records, they are currently unable to document counseling sessions or recommendations directly in the system. Consequently, these activities are recorded independently using self-developed formats. Although some CHCs are integrated into a centralized data system, others still rely on local databases. The current manual and disorganized documentation system presents a challenge, but future integration with electronic systems at CHCs could resolve this. Other studies have shown that information technology allows the entire healthcare team to identify and address drug therapy problems.^30^^,^^33^^,^^34^

MTM requires readiness in several areas, including skilled personnel, infrastructure, and stakeholder support. In this study, pharmacists emphasized the need for specialized MTM training. Intensive training with dedicated modules is essential for successful MTM implementation in CHCs. Additionally, resources such as counseling rooms, electronic service systems, and standardized forms are necessary. Research has shown that such training enhances pharmacists' knowledge and attitudes towards patient care.^28^^,^^35^

The success of MTM in CHCs depends on various factors, with time allocation for MTM services being key. Pharmacists often feel burdened by routine duties, leaving little time for patient communication and monitoring. Documentation and recording skills also pose challenges. Some studies suggest virtual MTM models and videoconferencing as potential solutions.^36^^,^^37^ Integration of MTM into existing pharmacy workflows is also recommended.^30^^,^^38^

Equally important is the support from management, other healthcare providers, and all CHC stakeholders. This support facilitates MTM adoption. Competency training in MTM is crucial and aligns with research from Indonesia, indicating that CHCs managers play a pivotal role in MTM's success by providing regulations, procedures, and program socialization.^26^

This study suggests some strategies to improve MTM services in Indonesia encompass several key steps. It is recommended that pharmacists enhance the competence of pharmacists and healthcare professionals through continuous education and MTM certification to ensure the necessary skills, foster closer interprofessional collaboration between pharmacists, doctors, and other healthcare providers within interdisciplinary teams, utilize technology such as digital platforms or telehealth to monitor and manage patient medication therapy in real-time, especially in remote areas. Pharmacists are also recommended to advocate for government policies that support the implementation of MTM through incentives and integration into the national health insurance system. Additionally, raising public awareness about MTM through educational campaigns is crucial, along with conducting regular monitoring and evaluation to ensure the ongoing effectiveness and improvement of MTM services. Future research should evaluate the effectiveness of strategies for improving MTM services in Indonesia, including the impact of continuous education, interprofessional collaboration, digital health technologies, and public policy integration, while also assessing the role of public awareness campaigns and ongoing monitoring in enhancing service quality and sustainability.

Although this study shares similar results with previous research in Indonesia, it introduces a novel approach by tailoring improvements to MTM services within CHCs, offering a dual-perspective analysis, and providing practical recommendations for overcoming specific implementation barriers. Additionally, it contributes to the literature by addressing gaps in MTM service implementation in CHCs and proposing solutions specifically designed for primary care settings in developing regions. Although this study does not examine the relationship between variables, it thoroughly investigates the implementation of MTM and its challenges. Furthermore, the study's findings are limited to CHCs in Yogyakarta, making it insufficient to generalize MTM services across all CHCs in Indonesia. However, these findings provide a foundation for further research and development of MTM in Indonesia and neighboring countries. In general, this study offers an overview of MTM practices in Indonesia.

## 5 Conclusion

From the perspectives of patients and pharmacists, this study shows that MTM services have not been fully implemented by pharmacists at CHCs. The services provided so far partially reflect the elements of MTM, although both groups of respondents believe that not all aspects have been carried out at these CHCs. On the positive side, both groups view the implementation of MTM as bringing many benefits. The future implementation of MTM is expected to be more adaptive to the conditions of the CHCs, integrated with existing systems, standardized in terms of human resources, procedures, and facilities, and supported by all parties at the CHCs.

## Ethics approval and consent to participate

Ethics permission for performing this study was granted by the Medical and Health Research Ethics Committee, Faculty of Medicine, Public Health, and Nursing, Universitas Gadjah Mada, Indonesia, with letter number KE/FK/1879/EC/2023. All participants provided informed consent before participating in the study.

## Consent for publication

Not applicable.

## Funding

This study was supported by the Faculty of Pharmacy and 10.13039/501100012521Universitas Gadjah Mada, Yogyakarta, Indonesia through a research grant. The sponsor had no role participated in the research concept, analysis, or final interpretation of data, including drafting the manuscript or deciding to publish the results.

## CRediT authorship contribution statement

**Nanang Munif Yasin:** Writing – review & editing, Writing – original draft, Validation, Supervision, Investigation, Formal analysis, Conceptualization. **Fivy Kurniawati:** Writing – review & editing, Writing – original draft, Methodology, Conceptualization. **Firda Ridhayani:** Writing – review & editing, Writing – original draft, Methodology, Conceptualization.

## Declaration of competing interest

The authors declare no conflicts of interest in this work.

## Data Availability

The datasets used and/or analyzed during the current study are available from the corresponding author upon reasonable request.

## Appendix A Supplementary data

COREQ Checklist Image 1
